# Generation of KCL039 clinical grade human embryonic stem cell line

**DOI:** 10.1016/j.scr.2015.12.036

**Published:** 2016-01

**Authors:** Liani Devito, Victoria Wood, Neli Kadeva, Glenda Cornwell, Stefano Codognotto, Emma Stephenson, Dusko Ilic

**Affiliations:** Stem Cell Laboratories, Division of Women's Health, Faculty of Life Sciences and Medicine, King's College London and Assisted Conception Unit, Guys'’ Hospital, London, United Kingdom

## Abstract

The KCL039 human embryonic stem cell line was derived from a normal healthy blastocyst donated for research. The ICM was isolated using laser microsurgery and plated on γ-irradiated human foreskin fibroblasts. Both the derivation and cell line propagation were performed in an animal product-free environment and under current Good Manufacturing Practice (cGMP) standards. Pluripotent state and differentiation potential were confirmed by in vitro assays.

## Resource table

**Table:** 

Name of stem cell line	KCL039
Institution	King's College London, London UK
Derivation team	Neli Kadeva, Victoria Wood, Glenda Cornwell, Stefano Codognotto, Emma Stephenson
Contact person and email	Dusko Ilic, email: dusko.ilic@kcl.ac.uk
Date archived/stock date	Feb. 03, 2012
Type of resource	Biological reagent: cell line
Sub-type	Human pluripotent stem cell line
Origin	Human embryo
Key marker expression	Pluripotent stem cell markers: NANOG, OCT4, TRA-1-60, TRA-1-81, alkaline phosphatase (AP) activity
Authentication	Identity and purity of line confirmed
Link to related literature (direct URL links and full references)	1) Jacquet, L., Stephenson, E., Collins, R., Patel, H., Trussler, J., Al-Bedaery, R., Renwick, P., Ogilvie, C., Vaughan, R., Ilic, D., 2013. Strategy for the creation of clinical grade hESC line banks that HLA-match a target population. EMBO Mol. Med. 5 (1), 10–17. doi: 10.1002/emmm.201201973 http://www.ncbi.nlm.nih.gov/pubmed/23161805 2) Canham, A., Van Deusen, A., Brison, D.R., De Sousa, P., Downie, J., Devito, L., Hewitt, Z.A., Ilic, D., Kimber, S.J., Moore, H.D., Murray, H., Kunath, T., 2015. The molecular karyotype of 25 clinical-grade human embryonic stem cells lines. *Sci. Rep.* 5, 17258. doi: 10.1038/srep17258 http://www.ncbi.nlm.nih.gov/pubmed/26607962 3) Ilic, D., Stephenson, E., Wood, V., Jacquet, L., Stevenson, D., Petrova, A., Kadeva, N., Codognotto, S., Patel, H., Semple, M., Cornwell, G., Ogilvie, C., Braude, P., 2012. Derivation and feeder-free propagation of human embryonic stem cells under xeno-free conditions. Cytotherapy. 14 (1), 122–128. doi: 10.3109/14653249.2011.623692 http://www.ncbi.nlm.nih.gov/pubmed/22029654 4) Stephenson, E., Jacquet, L., Miere, C., Wood, V., Kadeva, N., Cornwell, G., Codognotto, S., Dajani, Y., Braude, P., Ilic, D., 2012. Derivation and propagation of human embryonic stem cell lines from frozen embryos in an animal product-free environment. Nat. Protoc. 7 (7), 1366–1381. doi: 10.1038/nprot.2012.080 http://www.ncbi.nlm.nih.gov/pubmed/22722371
Information in public databases	KCL039 is a National Institutes of Health (NIH) registered hESC line NIH Registration Number: NIHhESC-14-0274 http://grants.nih.gov/stem_cells/registry/current.htm?id=680
Ethics	The hESC line KCL039 is derived under license from the UK Human Fertilisation and Embryology Authority (research license numbers: R0075 and R0133) and also has local ethical approval (UK National Health Service Research Ethics Committee Reference: 06/Q0702/90). Informed consent was obtained from all subjects and the experiments conformed to the principles set out in the WMA Declaration of Helsinki and the NIH Belmont Report. No financial inducements are offered for donation.

## Resource details

**Table:** 

Consent signed	Nov. 15, 2011
Embryo thawed	Jan. 16, 2012
UK Stem Cell Bank Deposit Approval	Reference: SCSC12-37
Sex	Male 46, XY
Grade	Clinical
Disease status	Healthy/Unaffected
Karyotype (aCGH)	No copy number changes detected
SNP array	No structural genomic variants detected (Canham et al., 2015)
DNA fingerprint	Allele sizes (in bp) of 16 microsatellite markers specific for chromosomes 13, 18 and 21 (Jacquet et al., 2013)
HLA typing	HLA-A 01, 24; B 35, 49; Bw 4, 6; C 04, 07; DRB1 03; DRB3 01/02; DQB1 02 (Jacquet et al., 2013, Canham et al., 2015)
Viability testing	Pass
Mycoplasma	Negative
Sterility	Pass
Pluripotent markers (immunostaining) (Fig. 1)	NANOG, OCT4, TRA-1-60, TRA-1-81, AP activity
Three germ layers differentiation in vitro (immunostaining) (Fig. 2)	Endoderm: AFP Ectoderm: TUBB3 (tubulin, beta 3 class III) Mesoderm: ACTA2 (actin, alpha 2, smooth muscle)
Sibling lines available	No

**Fig. 1:**
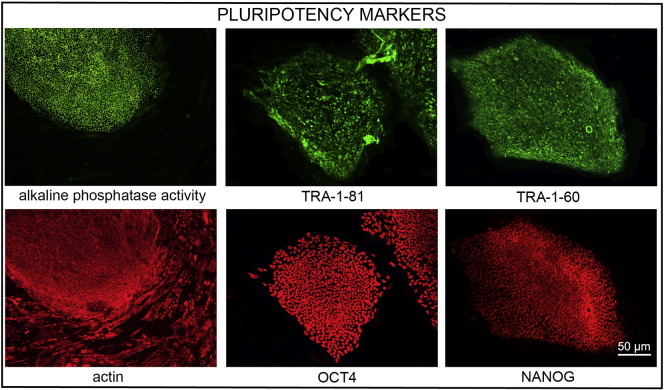
Expression of pluripotency markers. Pluripotency is confirmed by immunostaining (Oct4, Nanog, TRA-1-60, TRA-1-81) and alkaline phosphatase (AP) activity assay. Actin stress fibers, visualized with rhodamine-phalloidin (red), are present in both feeders and hES cell colonies, whereas AP activity (green) is detected only in hES cells. Scale bar, 50 μm.

**Fig. 2:**
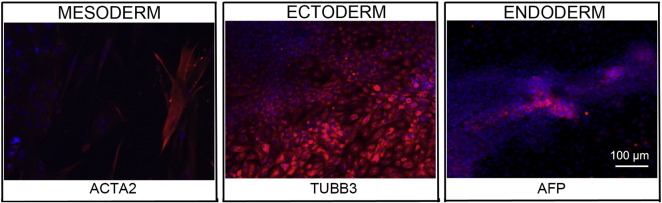
Differentiation of three germ layers in vitro is confirmed by detection of markers: smooth muscle actin (red) for mesoderm, β-III tubulin (red) for ectoderm and α-fetoprotein (red) for endoderm. Nuclei are visualized with Hoechst 33342 (blue). Scale bar, 50 μm.

We generated a KCL039 clinical grade hESC line following protocols, established previously (Ilic et al., 2012, Stephenson et al., 2012), and now adapted to cGMP conditions. The expression of the pluripotency markers was tested after a freeze/thaw cycle (Fig. 1). Differentiation potential into three germ layers was verified in vitro (Fig. 2).

The embryo donors were negative for Human Immunodeficiency Virus 1/2 (HIV1/2), Hepatitis B (HepB, HCB) and C Virus (HepC, HCV). We did not retest the line.

We also generated a research grade of the KCL039 line that is adapted to feeder-free conditions.

## Materials and methods

### Consenting process

We distributed patient information sheets (PIS) and consent forms to the in vitro fertilization (IVF) patients if they opted to donate to research embryos that were stored for 5 or 10 years. They mail signed consent back to us and that might be months after the PIS and consent forms were mailed to them. If in the meantime new versions of PIS/consent forms are implemented, we do not send these to the patients or ask them to re-sign; the whole process is done with the version that was given them initially. The PIS/consent documents (FRO-V.10) were created on Sep. 19, 2011. The HFEA Code of Practice that was in effect at the time of document creation was: Edition 8 — R.3 (http://www.hfea.gov.uk/2999.html). The donor couple signed the consent on Nov. 15, 2011. The HFEA Code of Practice that was in effect at the time of donor signature was: Edition 8 — R.4. The HFEA Code of Practice Edition 8 — R.3 was in effect from Apr. 07, 2011 to Oct. 01, 2011, whereas 8 — R.4 was in effect from Oct. 02, 2011 to Apr. 02, 2012.

### Embryo culture and micromanipulation

Embryo culture and laser-assisted dissection of inner cell mass (ICM) were carried out as previously described in details (Ilic et al., 2012, Stephenson et al., 2012). The cellular area containing the ICM was then washed and transferred to plates containing mitotically inactivated human neonatal foreskin fibroblasts (HFF).

### Cell culture

ICM plated on mitotically inactivated HFF were cultured as previously described (Ilic et al., 2012, Stephenson et al., 2012). Trophectoderm cells were removed mechanically from outgrowth (Ilic et al., 2007, Ilic et al., 2010). hESC colonies were expanded and cryopreserved at the third passage.

### Viability test

Straws with the earliest frozen passage (p. 2–3) are thawed and new colonies are counted three days later. These colonies are then expanded up to passage 8, at which point cells were partly frozen and partly subjected to a standard battery of tests (pluripotency markers, in vitro and in vivo differentiation capability, genetics, sterility, and mycoplasma).

### Pluripotency

Pluripotency in vitro was assessed using two different techniques: enzymatic activity assay [alkaline phosphatase (AP) assay] and immunostaining as previously described (Ilic et al., 2012, Stephenson et al., 2012).

### Differentiation

Spontaneous differentiation into three germ layers was assessed in vitro as previously described (Petrova et al., 2014, Stephenson et al., 2012).

### Genotyping

DNA was extracted from hESC cultures using a Chemagen DNA extraction robot according to the manufacturer's instructions. Amplification of polymorphic microsatellite markers was carried out as previously described (Ilic et al., 2012). Allele sizes were recorded to give a unique fingerprint of each cell line.

### Array comparative genomic hybridization (aCGH)

aCGH was performed as described in detail by Ilic et al. (2012).

### Whole-genome single nucleotide polymorphism (SNP) array

SNP array was performed as described in detail by Canham et al. (2015).

### HLA typing

HLA-A, -B and -DRB1 typing was performed with a PCR sequence-specific oligonucleotide probe (SSOP; Luminex, Austin, TX, USA) hybridization protocol at the certified Clinical Transplantation Laboratory, Guy's and St Thomas' NHS Foundation Trust and Serco Plc. (GSTS) Pathology (Guy's Hospital, London, UK) as previously described (Jacquet et al., 2013). HLA typing was also performed independently by another group (Canham et al., 2015).

## Author disclosure statement

There are no competing financial interests in this study.
